# Global burden of occupational ergonomic factor-induced low back pain, 1990~2021: data analysis and projections of the global burden of disease

**DOI:** 10.3389/fpubh.2025.1573828

**Published:** 2025-05-27

**Authors:** Meng Wu, Peihong Wu, Huaye Lu, Lei Han, Xin Liu

**Affiliations:** ^1^Institute of Occupational Disease Prevention, Jiangsu Provincial Center for Disease Control and Prevention, Nanjing, China; ^2^NHC Key Laboratory for Engineering Control of Dust Hazard, Beijing, China

**Keywords:** disease burden, low back pain, occupational ergonomic factors, estimate projections, epidemiology

## Abstract

**Background:**

Low back pain (LBP) is a global epidemic that severely affects the quality of life and imposes a substantial economic burden worldwide. Occupational ergonomic factors are the most important modifiable contributors to LBP. In this study, we estimated the global burden of occupational ergonomic factor-induced LBP from 1990 to 2021 using the Global Burden of Disease, Injuries, and Risk Factors Study (GBD) 2021 database and projected future trends.

**Methods:**

Global years lived with disability (YLDs) and age-standardized YLD rate (ASYLDR) for occupational ergonomic factor-induced LBP by sex and Socio-demographic index (SDI) among individuals aged 15–64 from 1990 to 2021 were obtained from the GBD 2021. Long-term trends were evaluated by calculating the average annual percent change (AAPC) of ASYLDR using a Joinpoint model. A Nordpred model was applied to analyze temporal changes in overall and age-specific YLDs and ASYLDR between 1990 and 2021, and to project trends from 2022 to 2045.

**Results:**

From 1990 to 2021, global YLDs of occupational ergonomic factor-induced LBP increased by 40.63%. Projections indicated that all-age YLDs will exceed 15 million person-years by 2037. The ASYLDR of occupational ergonomic factor-induced LBP had shown a sustained decline since 1990, decreasing by 18.75% between 1990 and 2021, with an AAPC of −0.670% (95% confidence interval: −0.718 to −0.622). This downward trend is expected to persist until 2045. The SDI showed a negative correlation with ASYLDR (*R* = −0.36, *p* < 0.001). YLDs and ASYLDR were consistently higher in females than in males.

**Conclusion:**

While the disease burden of occupational ergonomic factor-induced LBP has decreased, the reduction remains modest. Females, low SDI regions, and middle-aged/older adults (40–64 years) are the main contributors to the disease burden. Occupational ergonomic factor-induced LBP remains a critical public health problem that requires urgent attention to find global, comprehensive, effective, and targeted prevention strategies.

## Introduction

Low back pain (LBP) is one of the common musculoskeletal disorders (MSDs) with a global prevalence (1). According to the Global Burden of Disease, Injuries, and Risk Factors Study (GBD) 2021 study, the number of prevalent cases attributable to LBP increased significantly between 1990 and 2021, with over 600 million people affected globally in 2020 (2). Moreover, LBP ranks first among causes affecting global years lived with disability (YLDs) counts and age-standardized rates, is the largest contributor to global, all-cause, male, and female YLDs in 2021, and remains a major driver of disability-adjusted life years (DALYs) globally (2). By contributing to disability, LBP severely impairs global productivity and quality of life while placing a substantial economic burden on countries. Therefore, comprehensive monitoring of the global disease burden attributable to LBP is essential.

The three modifiable risk factors contributing to the global burden of LBP are occupational ergonomic factors, smoking and elevated body mass index. Among these, occupational ergonomic factors have the greatest impact, with nearly one-quarter being attributable to LBP cases (3), and increasing the risk of suffering LBP (4). Occupational ergonomic factors include exertion, difficult postures, repetitive motions, hand vibration, heavy lifting, kneeling and/or squatting, and climbing (5). A study established a causal relationship between occupational factors (heavy labor) and LBP development (6). While technological advances lead to sedentary work styles (7), this shift may paradoxically increase chronic LBP incidence. Currently, previous studies have utilized the GBD database to examine the global prevalence trends and the burden of LBP, while few research has been conducted on LBP attributable to occupational ergonomic factors.

In this paper, we estimated the global burden of LBP attributable to occupational ergonomic factors across 204 countries and territories from 1990 to 2021, assessing both all-age YLDs and age-standardized YLD rate (ASYLDR). Our primary objective was to evaluate long-term trends in occupational ergonomic factor-induced LBP within the GBD framework. Furthermore, we conducted a stratified analysis to examine variations in disease burden by sex, age group, and Socio-demographic index (SDI). To project future trends, we applied Joinpoint regression to analyze temporal patterns and the Nordpred age-period-cohort model to forecast YLDs and ASYLDR up to 2045. This comprehensive approach provides insights into the disease burden of occupational ergonomic factor-induced LBP and informs global prevention strategies.

## Methods

### Data sources

The GBD database integrates heterogeneous data from different countries with different qualities, ensures global comparability and the reliability of research results by adopting a standardized methodological framework [including Bayesian statistics and DisMod-MR (disease model-Bayesian meta-regression) models] to address data gaps and heterogeneity (2). The transparency and reproducibility of GBD methodology have established it as the gold standard for GBD research.

Data for this study were obtained from the GBD 2021 database provided by the Institute for Health Metrics and Evaluation (IHME), and data input sources included censuses, vital statistics, surveys, and other health-related data sources. Through the Global Health Data Exchange (GHDx) platform,^1^ we obtained YLDs and ASYLDR for LBP induced by occupational ergonomic factors among individuals aged 15–64 years globally between 1990 to 2021.

### Estimate the disease burden of occupational ergonomic factor-induced LBP

LBP is defined as pain that lasts for at least one day in the posterior region of the body from the lower edge of the twelfth rib to the subgluteal crease, with or without pain directed to one or both lower extremities (3). As a nonfatal outcome, LBP data were derived from published studies, surveys, notification data, disease registries, or hospital inpatient data. Disease prevalence was estimated by sex, age, location, and year using DisMod-MR 2.1, a Bayesian meta-regression tool that ensures internal consistency among incidence, prevalence, and mortality data (8). Prevalence was multiplied by the disability weight of the health condition associated with the sequelae to calculate the YLDs. 1,000 estimates were generated from the model, with 95% uncertainty intervals (UI) representing the distribution’s 25th-975th values.

### SDI

The SDI is a composite measure widely used in burden of disease research, which consists of the mean educational attainment among individuals aged ≥15 years, the total fertility rate of the population under 25 years, and the per capita lagged distribution income (3). As a comprehensive development indicator, SDI reflects both socioeconomic conditions and health system capacity. The standardized calculation of the SDI facilitates robust global comparisons and demonstrates strong predictive validity for health disparities across development spectrums, particularly for critical outcomes like morbidity and mortality rates. In the 2021 update (2), the GBD study refined SDI report by transforming the original 0–1 scale to a percentage metric (multiplied by 100) and establishing five development tiers based on contemporary data: low SDI (0–45.5), low-middle SDI (45.6–60.8), middle SDI (60.9–69.0), high-middle SDI (69.1–80.5), and high SDI (80.6–100).

### Statistical analysis

#### The average annual percent change for ASYLDR

The Joinpoint model plays an important role in disease trend analysis, automatically identifying turning points in morbidity or mortality trends. This approach is particularly valuable for detecting trend transitions and evaluating the impact of policy interventions. In addition, the model outputs annual percent change metrics that visualize the rate of change in trends, which are easy for public health decision makers to understand and apply (9).

ASYLDR per 100,000 population is shown with 95% UI. To analyze long-term trends in ASYLDR for occupational ergonomic factor-induced LBP, as previously described (10), we calculated the average annual percent change (AAPC) and the corresponding 95% confidence interval (CI) for ASYLDR using Joinpoint regression software (V.4.7.0.0) by dividing the study period into six intervals defined by five optimal joinpoints, and performing separate trend fitting and optimization for each segment.

#### Estimates of projections

The Nordpred model is a widely utilized epidemiological tool for analyzing and forecasting disease trends, with its principal strength lying in its ability to decompose temporal patterns into age, period, and cohort effects, a critical feature for elucidating long-term dynamics of disease burden (11).

Conventional approaches exhibit notable limitations: time-series models (e.g., ARIMA) fail to account for cohort effects (12); machine learning methods (e.g., LSTM) are prone to overfitting short-term fluctuations while lacking interpretability (13); and traditional regression performs poorly with sparse data. In contrast, Nordpred demonstrates exceptional robustness in long-term projections, particularly for forecasts spanning 25 + years, as it operates under the assumption of historical trend continuity (14). This makes it especially suitable for modeling the progression of chronic diseases or cancer incidence, a capability that has established it as a gold-standard methodology for public health planning.

In this study, a Nordpred model based on the GBD 2021 data was developed using R software (version 4.0.3) to characterize the temporal changes in ASYLDR of occupational ergonomic factor-induced LBP. The analysis examined global patterns and age-specific trends from 1990 to 2021, with projections extended through 2045.

#### Correlation analysis

The relationship between ASYLDR of occupational ergonomic factor-induced LBP and SDI across 204 countries and territories in 2021 was analyzed, and the strength of the correlation was measured by the Pearson correlation coefficients. All analyses and trend modeling were conducted using R software (version 4.0.3).

## Results

### Trends in the burden of low back pain due to occupational ergonomic factors, globally, by SDI and age

**Figure 1:**
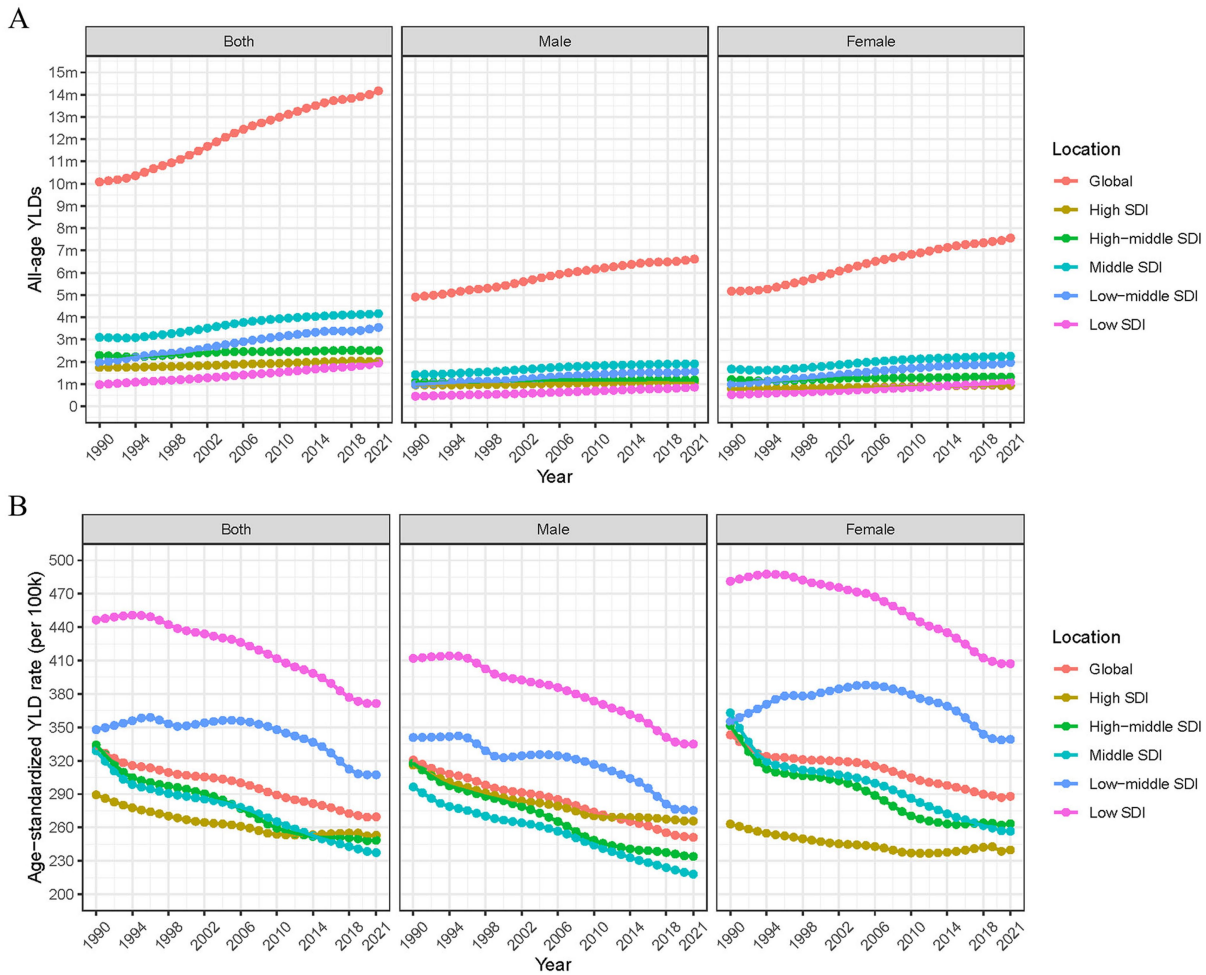
Regional and gender-specific changes in all-age YLDs and age-standardized YLD rate for occupational ergonomic factor-induced low back pain, 1990–2021. **(A)** All-age YLDs. **(B)** Age-standardized YLD rate (per 100 k). YLDs, years lived with disability; SDI, socio-demographic index.

We analyzed the number and trends of YLDs attributable to occupational ergonomic factor-induced LBP across 204 countries and territories between 1990 and 2021. The results showed a steady annual increase in global YLDs of occupational ergonomic factor-induced LBP, rising from 10.078 million (95% UI: 5.969–15.551) in 1990 to 14.173 million (8.466–21.906) in 2021, reflecting a 40.63% increase (Table 1; Figure 1A). YLDs were slightly higher in females than in males (Figure 1A). During the same period, the global ASYLDR exhibited an initial increase followed by a decline, peaking in 1994 before decreasing annually (Figure 1B). The rate declined from 331.696 (196.914–512.356) per 100,000 population in 1990 to 269.504 (160.914–416.487) per 100,000 population in 2021, marking an 18.75% reduction (Table 1).

**Table 1:** Changes in all-age YLDs, age-standardized YLD rate for occupational ergonomic factor-induced LBP in 1990 and 2021.

Location	All-age YLDs			Age-standardized YLD rate (per 100 k)			
	1990, millions (95% UI)	2021, millions (95% UI)	Percent change, 1990–2021	1990 (95% UI)	2021 (95% UI)	Percent change, 1990–2021	AAPC, % (95% CI)
Global	10.078 (5.969 ~ 15.551)	14.173 (8.466 ~ 21.906)	40.63	331.696 (196.914 ~ 512.356)	269.504 (160.914 ~ 416.487)	−18.75%	−0.670 (−0.718 ~ −0.622)
High-middle SDI	2.288 (1.353 ~ 3.534)	2.503 (1.497 ~ 3.854)	9.40	334.172 (197.894 ~ 516.578)	248.526 (148.236 ~ 382.754)	−25.63%	−0.434 (−0.482 ~ −0.386)
High SDI	1.746 (1.06 ~ 2.633)	2.018 (1.256 ~ 2.983)	15.58	289.364 (175.563 ~ 436.333)	253.032 (157.406 ~ 374.022)	−12.56%	−0.942 (−1.008 ~ −0.875)
Low-middle SDI	1.962 (1.139 ~ 3.067)	3.542 (2.063 ~ 5.559)	80.53	347.879 (202.951 ~ 543.915)	307.381 (179.406 ~ 482.394)	−11.64%	−1.053 (−1.094 ~ −1.013)
Low SDI	0.974 (0.572 ~ 1.508)	1.935 (1.136 ~ 3.011)	98.67	446.425 (263.26 ~ 691.694)	371.431 (219.183 ~ 578.179)	−16.80%	−0.400 (−0.451 ~ −0.349)
Middle SDI	3.097 (1.803 ~ 4.82)	4.163 (2.453 ~ 6.454)	34.42	328.897 (191.947 ~ 512.408)	237.436 (140.015–368.11)	−27.81%	−0.595 (−0.628 ~ −0.563)

YLDs, years lived with disability; UI, uncertainty intervals; AAPC, average annual percent change; CI, confidence interval.

The YLDs of occupational ergonomic factor-induced LBP increased across all SDI regions between 1990 and 2021. Moreover, YLDs rose with higher SDI levels, with the most significant increase occurring in low-middle SDI regions (Table 1; Figure 1A). In addition, ASYLDR was significantly higher in low SDI regions than in other SDI groups (Figure 1B). Gender disparities were also observed, with females exhibiting higher ASYLDR than males in all SDI groups except high SDI regions (Figure 1B). Over the 32-year period, female ASYLDR fluctuations were most prominent in low-middle SDI regions, characterized by an annual increase from 1990 to 2005, followed by a gradual decline thereafter (Figure 1B).

### Temporal changes in the burden of low back pain due to occupational ergonomic factors, globally and by SDI

**Figure 2:**
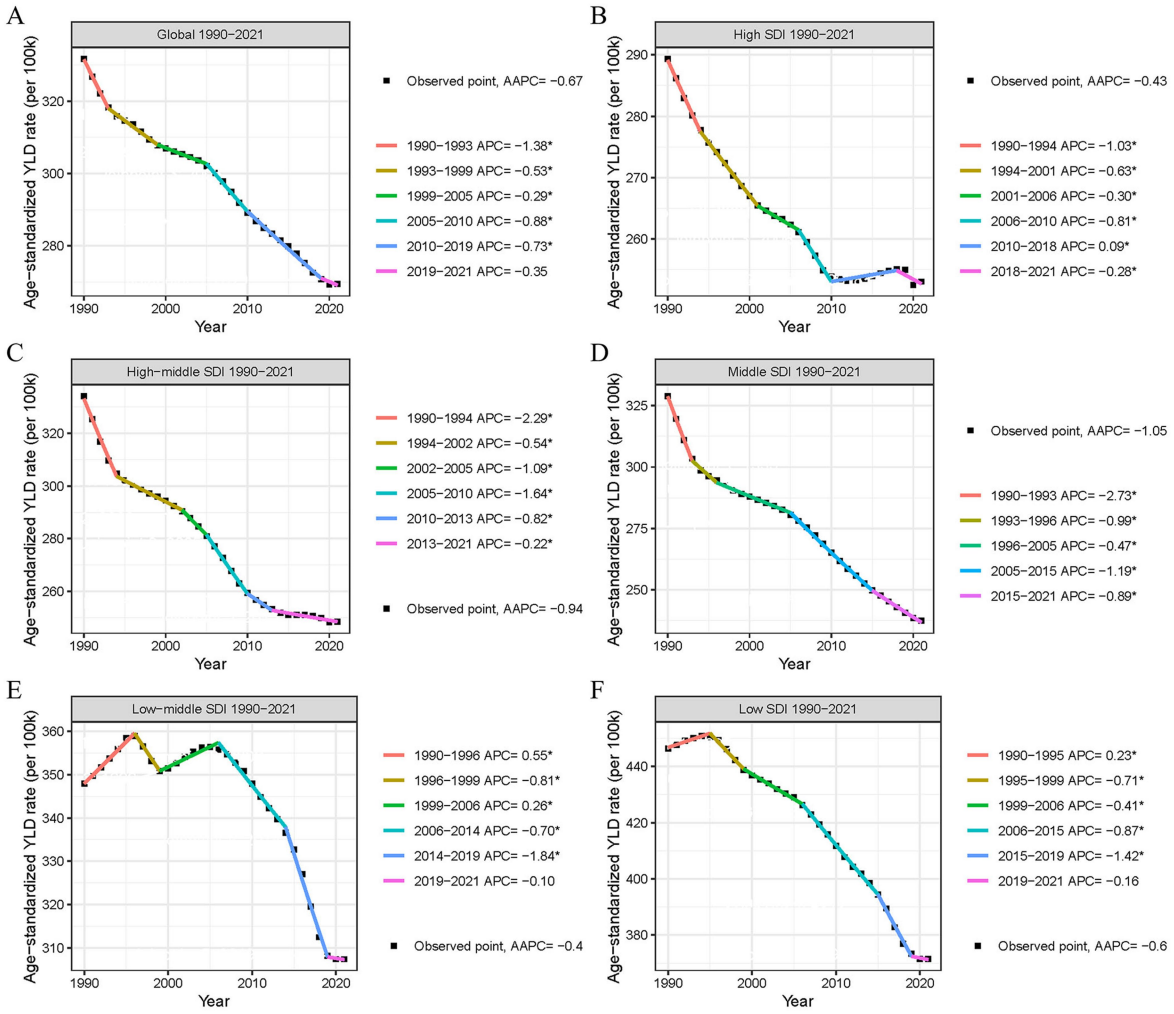
Changes in age-standardized YLD rate for occupational ergonomic factor-induced low back pain, globally and in different SDI regions, 1990–2021. **(A)** Global. **(B)** High SDI region. **(C)** High-middle SDI region. **(D)** Middle SDI region. **(E)** Low-middle SDI region. **(F)** Low SDI region. YLDs, years lived with disability; SDI, socio-demographic index.

We used the Joinpoint model to calculate the AAPC of ASYLDR for occupational ergonomic factor-induced LBP from 1990 to 2021, and to assess the temporal trends. Globally, between 1990 and 2021, ASYLDR exhibited an overall decline (AAPC < 0, *p* < 0.05) across all SDI regions, though trends varied. The global AAPC was −0.670% (95% CI: −0.718% to −0.622%) (Table 1), with turning points in 1993, 1999, 2005, 2010, and 2019 (Figure 2A). The steepest decline (−1.38%) occurred between 1990 and 1993 (Figure 2A). ASYLDR in the high-middle and middle SDI regions showed sustained decreases, with the largest decreases in 1990–1994 and 1990–1993, respectively (Figures 2C,D). High SDI regions experienced an increase in ASYLDR (AAPC: 0.09%) during 2010–2018 (Figure 2B). Low-middle SDI regions displayed an “M” shaped, peaking in 1996 and 2006, with the sharpest rise in 1990–1996 and the steepest decline in 2014–2019 (Figure 2E). Low SDI regions saw an initial increase (AAPC: 0.23%) from 1990 to 1995, followed by a sustained decrease, with the greatest drop in 2015–2019 (Figure 2F).

### Distribution of low back pain burden due to occupational ergonomic factors by location, 1990 and 2021

**Figure 3:**
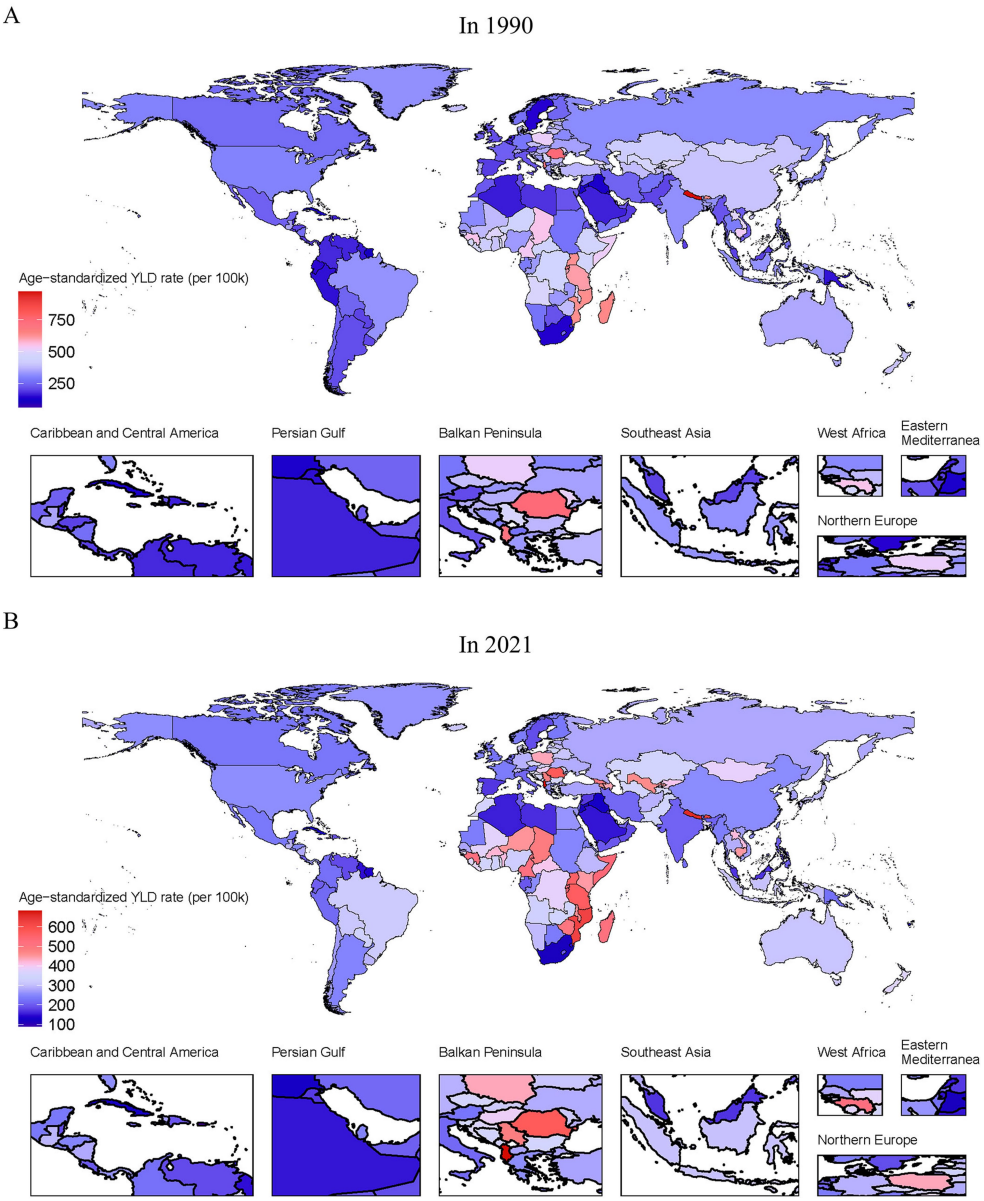
Global age-standardized YLD rate for occupational ergonomic factor-induced low back pain in 1990 and 2021. **(A)** In 1990. **(B)** In 2021.

Figures 3A,B and Supplementary material present the global ASYLDR of occupational ergonomic factor-induced LBP in 1990 and 2021. In 1990, Nepal showed the highest ASYLDR (971.742 per 100,000 population; 95% UI: 586.965–1487.809), followed by Romania (745.706, 435.855–1163.225) (Figure 3A; Supplementary material). The lowest rates occurred in Kiribati (61.664, 31.843–105.495) and Puerto Rico (96.184, 56.674–148.961) (Figure 3A; Supplementary material). By 2021, Albania had the highest ASYLDR (688.222, 388.186–1100.363), with Nepal ranking second (657.884, 369.498–1042.279), while Puerto Rico (88.720, 49.721–143.021) and Fiji (114.777, 65.269–180.784) demonstrated the lowest rates (Figure 3B; Supplementary material). Notably, Nepal experienced the greatest percentage decrease in ASYLDR (−32.3%), declining from 971.742 (586.965–1487.809) to 657.88 (369.498–1042.279) per 100,000 population, whereas the Solomon Islands showed the most dramatic increase (171.4%), rising from 115.54 (60.48–194.43) to 313.58 (180.25–495.21) per 100,000 population during this period (Supplementary material).

### Correlation between the burden of low back pain due to occupational ergonomic factors and SDI

**Figure 4:**
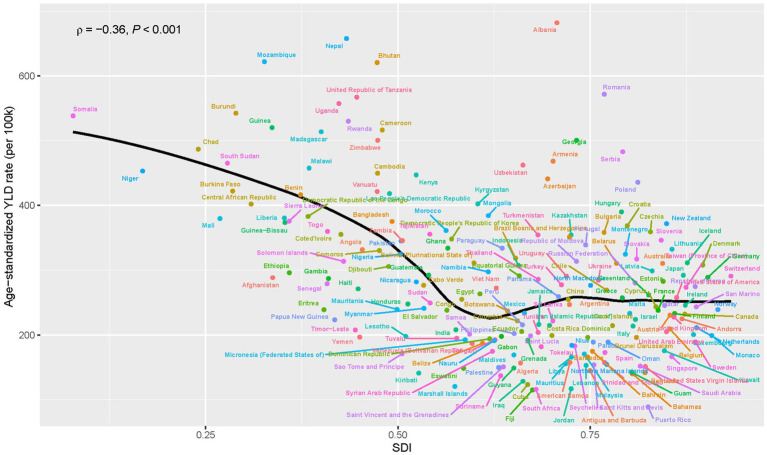
Relationship between age-standardized YLD rate and SDI for occupational ergonomic factor-induced low back pain, globally, 2021. Pearson correlation coefficients and *p*-values are shown. YLDs, years lived with disability; SDI, socio-demographic index.

Based on the differences in LBP burden across SDI regions, the correlation between ASYLDR and SDI was explored. The result showed a significant inverse correlation between ASYLDR and SDI across 204 countries (Pearson’s *R* = −0.36, *p* < 0.001; Figure 4). The analysis demonstrated an inverse relationship, wherein countries with lower SDI consistently exhibited higher ASYLDR, indicating a greater disease burden from occupational ergonomic factor-induced LBP.

### Distribution of low back pain burden due to occupational ergonomic factors, by sex and age, 1990 and 2021

**Figure 5:**
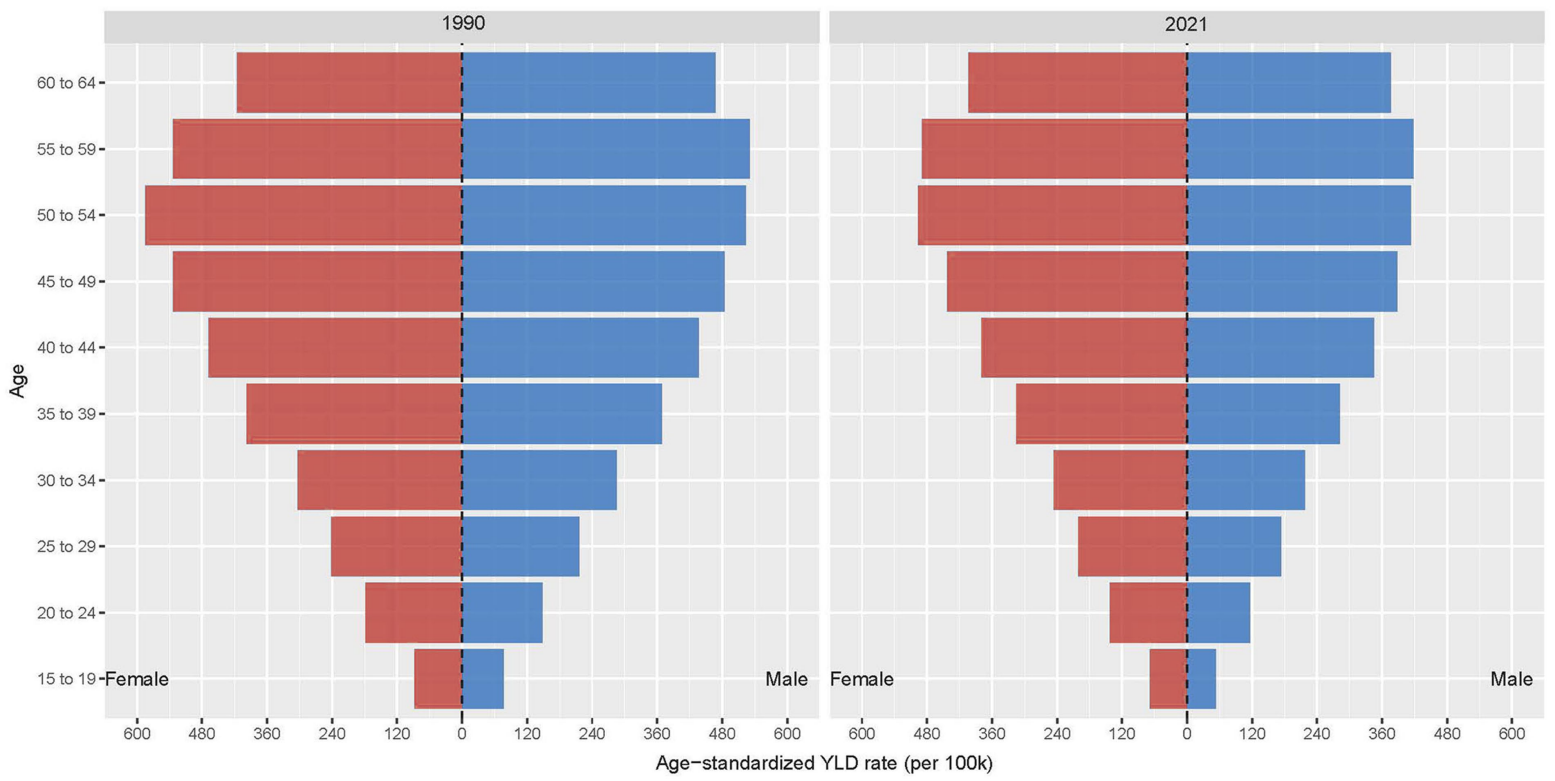
Global burden of occupational ergonomic factor-induced low back pain, by sex and age group, 1990 and 2021.

Figure 5 showed the burden of occupational ergonomic factor-induced LBP stratified by sex and age groups in 1990 and 2021. In terms of gender, females exhibited significantly higher ASYLDR than males across all age groups and time points. In terms of age, peak burden occurred in the 50–54-year age group for females and 55–59 years for males, and middle-aged and older age groups showed markedly higher ASYLDR compared to younger age group, and the 45–59-year age range represented the most severely affected demographic. In addition, we found that the sex disparity in disease burden widened over time, with females experiencing progressively greater relative impact (Figure 5).

### Global burden and prediction of occupational ergonomic factor-induced LBP by sex, from 1990 to 2045

**Figure 6:**
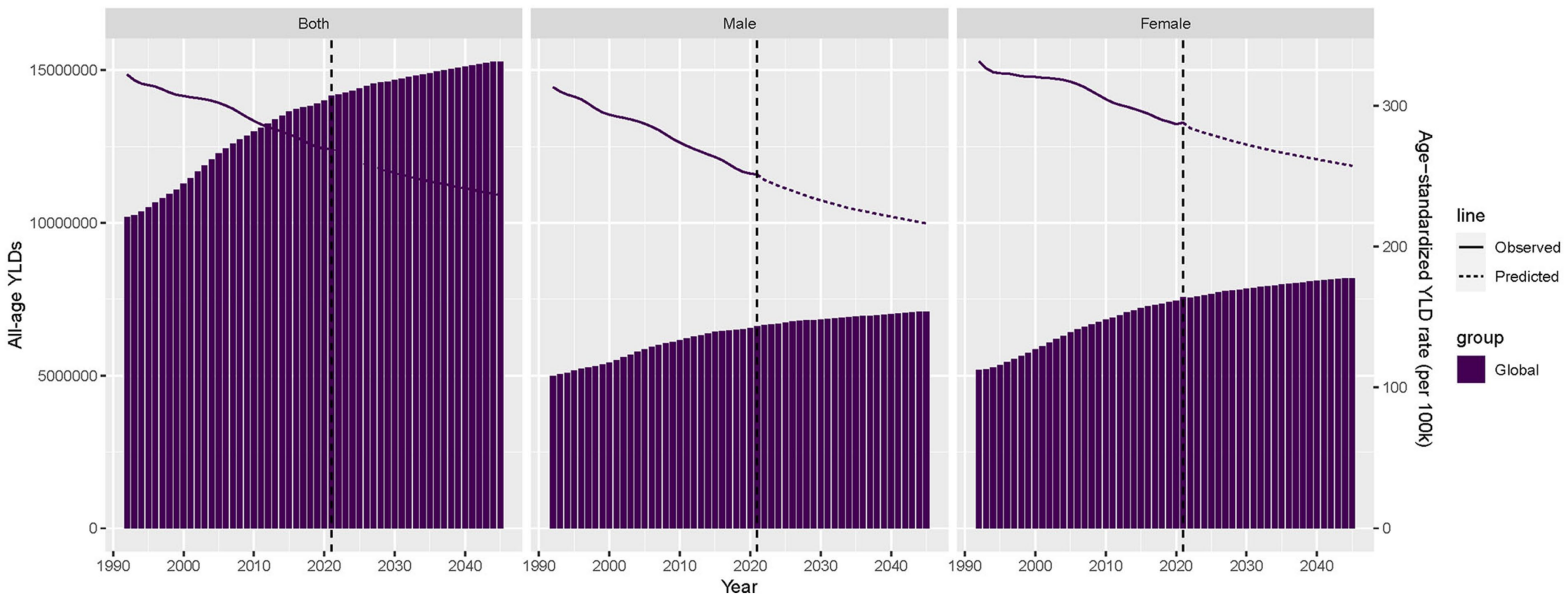
Trends and projections in all-age YLDs and age-standardized YLD rate of occupational ergonomic factor-induced low back pain, globally and by sex, 1990–2045. YLDs, years lived with disability.

We evaluated the all-age YLDs, ASYLDR, and projected trends of occupational ergonomic factor-induced LBP from 1990 to 2045. The ASYLDR had declined globally and by sex since 1990, with the downward trajectory projected to continue through 2045, and ASYLDR remained consistently higher in females than in males (Figure 6). In addition, the YLDs of occupational ergonomic factor-induced LBP had increased in all age from 1990 to 2045, and the global YLDs were projected to surpass 15 million person-years by 2037 (Figure 6). In terms of gender, YLDs also continued to increase in both sexes, and females maintain consistently higher YLD burdens than males (Figure 6).

## Discussion

DALYs reflect the overall burden of disease by measuring how diseases and injuries reduce healthy life years, which are composed of YLDs and years of life lost (2). The distribution of DALYs due to occupational ergonomic factor-induced LBP is dominated by YLDs. In this study, secondary data analysis was conducted using the GBD 2021 database to estimate and analyze the global burden of LBP attributable to occupational ergonomic factors from 1990 to 2021. The findings suggested that the disease burden of occupational ergonomic factor-induced LBP has been decreasing globally, especially in middle- and high-middle SDI regions. We estimated that the global ASYLDR decreased by 18.75% from 1990 to 2021, and by 2021, 269.504 per 100,000 population of the global ASYLDR for LBP was attributed to occupational ergonomic factors. Low SDI regions continued to have the highest disease burden, with middle-aged and older adults, as well as females, remained disproportionately affected.

### Clinical features and disease burden of LBP

LBP represents a clinical symptom that may result from several known or unknown abnormalities or diseases, most of which are self-limited with no specific cause and faster recovery (15). However, LBP is prone to repeated attacks (16), leading to long-term pain and even disability, and seriously affecting personal work ability and performance. This results in reduced productivity and huge economic losses for society (15, 17). At the individual level, persistent pain and disability associated with LBP can diminish quality of life, forcing early departure from work (16, 18), thus affecting survival and livelihood. According to the GBD 2021 estimates, the global prevalence of LBP in 2021 was 629 (552–701) million, an increase of 20.04% since 2010. The all-age YLDs was 70.2 (50.2–94.1) million person-years, and the ASYLDR was 832.2 (595.9–1115.3) per 100,000 population (2). Although the ASYLDR has declined since 2010, the reduction was only 2.37%, average 0.22% per year. Given its high prevalence, recurrence nature, and debilitating symptoms, LBP is increasingly recognized as a critical public health concern.

### Occupational risk factors

The latest monitoring data from the European Union reveals the severe situation of work-related MSDs. Among workers reporting occupational health problems, 60% list MSDs as the primary health concern (19). This public health challenge has been exacerbated by the ongoing digital transformation of labor markets. While technological advancements have reduced conventional physical demands, they have simultaneously introduced novel risk factors, including prolonged computer use and sustained static postures, characteristic of modern work patterns (20). Among various MSDs, LBP represents a particularly significant condition due to its high prevalence and substantial disability burden. The prevalence of LBP as a prevalent condition is as high as 67% among working population (16). Jacson et al. found that workers in low- and middle-income countries had a 2.5-fold higher risk of chronic LBP compared to non-working population (21). LBP ranks as the second most common reason for clinical visits and the leading cause of work**-**related disability (22). Occupational ergonomic factors, which are causally linked to LBP, represent significant contributors to the global rise in YLDs (3). Several studies demonstrated that weight lifting, stooping, hunching, awkward postures, and high physical labor at work increase the risk of developing LBP (23–26). We found that the global YLDs of occupational ergonomic factor-induced LBP increased steadily between 1990 and 2021, and the overall global burden was estimated to be about 14.173 million person-years in 2021. Projections indicate YLDs will surpass 15 million person-years by 2037.

### High-risk populations

Occupational ergonomic factor-induced LBP affects individuals across all age, including children, young adults, middle-aged adults and older adults. From 1990 to 2021, the global YLDs of occupational ergonomic factor-induced LBP increased by 40.63%, mainly driven by population aging and growth rather than an actual rise in incidence (27, 28). Over this 32-year period, the ASYLDR of occupational ergonomic factor-induced LBP exhibited an age**-**dependent increase, peaking in the 50–54-year age group. In addition, the ASYLDR also remained elevated among older workers (55–54 years). These are consistent with global trends in the age epidemiology of LBP. Over time, the global prevalence and severity of LBP have increased, with the incidence rising sharply in adolescence (29, 30). Recent epidemiological evidence indicates that 37% of adolescents experience LBP monthly or more frequently, and adolescent LBP significantly elevates the risk of persistent LBP in adulthood (31). It is estimated that the percentage of workers aged 50–64 will increase significantly in the future due to population aging (32). An epidemiologic study also found that the prevalence of LBP peaks at the age of 60–65 (33). However, although the incidence and prevalence of LBP is high in both adolescents and older adults, they are not usually included in LBP intervention studies (34), which is precisely the problem faced by occupationally exposed populations, where there is uncertainty about how to manage this population.

### Gender disparities

Globally, females had slightly higher YLDs for occupational ergonomic factor-induced LBP than males, with a greater proportion of females suffered from LBP. The ASYLDR was significantly higher in females than in males in all SDI groups except high SDI regions. By different age groups, females had higher ASYLDR than males in all groups. Existing literature reports that females experience more severe LBP manifestations, including higher prevalence rates, greater disability levels, and more complex complications (35–37). Occupational and ergonomic factors have been strongly associated with the development of LBP in women, including weight lifting operations, forward-leaning postures (both standing and sitting), and strenuous operations (17, 38). However, other studies have reported contrasting findings, such as Hooftman et al. and Aasa et al. found that men were at a higher risk of developing LBP when exposed to work-related risk factors compared to women (39, 40). The observed disparities reflect the complex, multidimensional nature of gender-specific patterns in occupational LBP. Societal gender roles likely expose women to dual ergonomic stressors, both occupational and domestic (17), creating a distinctive cumulative injury profile. Furthermore, female-dominated occupations typically involve sustained low-intensity loading exposures, while male-dominated fields are characterized by intermittent high-intensity demands. Biomechanically, women’s unique lumbopelvic characteristics confer greater susceptibility to repetitive loading (41). Sociocultural factors influence disease presentation through dual mechanisms. Gender norms suppress symptom reporting in men, while women’s greater healthcare utilization may partially amplify observed epidemiological differences (42). These findings demonstrate the urgent need to develop gender-sensitive assessment frameworks that integrate both occupational and domestic exposure metrics, along with sex-specific preventive interventions, for more accurate evaluation and management of gender disparities in occupational LBP.

### Socio-economic disparities

The SDI integrates key socio-economic indicators, including per capita income, educational attainment and fertility rates, to comprehensively assess regional development levels (2). SDI stratification serves as a valuable tool for identifying inequitable allocation of health resource and informing targeted policy interventions. In the present study, the YLDs of occupational ergonomic factor-induced LBP was highest in the middle SDI regions and the ASYLDR was highest in the low SDI regions in 1990 and 2021. From 1990 to 2021, as the SDI increased, the YLDs of occupational ergonomic factor-induced LBP also increased, especially in low- and low-middle SDI regions. Notably, over this 32-year period, Solomon Islands, a low-middle SDI region, experienced the most pronounced increase in disease burden. This trend likely stems from the nation’s substantial economic dependence on high-risk industries such as agriculture, forestry, and fisheries (43, 44). Workers in these sectors are exposed to prolonged heavy manual labor, compounded by inadequate occupational safeguards and healthcare deficiencies (45). This study also revealed a significant negative correlation between SDI and ASYLDR of occupational ergonomic factor-induced LBP. The elevated disease burden of LBP in low- and middle SDI regions is associated with weak enforcement of occupational health regulations, high prevalence of physically demanding labor, and insufficient ergonomic interventions in workplaces (15, 27, 46). Interestingly, certain middle- and high SDI regions (e.g., Romania) exhibit similarly high LBP burdens. This phenomenon may reflect imbalanced occupational transitions, where manufacturing sectors retain labor-intensive practices while emerging service industries fail to mitigate sedentary-related risks (47). Additional contributors include delayed occupational health policy implementation and workforce aging (2, 15). This SDI-stratified disparity underscores the need for tailored occupational health policies, low-SDI regions require strengthened foundational workplace protections, whereas high-SDI regions must prioritize interventions targeting sedentary behavior.

### Limitations and interventions

Although we have gained some insight into the GBD and its temporal trends in occupational ergonomic factor-induced LBP, several important limitations should be acknowledged. First, the predictive models failed to account for methodological constraints in GBD estimation, including heterogeneity in data sources and quality, as well as the impacts of COVID-19 on treatment accessibility and mortality rates in older adults. Notably, the widespread adoption of remote work during the COVID-19 pandemic introduced novel home ergonomic risks (e.g., non-standard working postures and improper equipment use) that remain systematically unevaluated (48). Second, most of the research evidence on LBP is provided by high-income countries, and its applicability to low- and middle-income countries is uncertain (15). Third, LBP is a non-communicable disease, and limited medical interventions in middle- and low-income countries will be more focused on priorities such as infectious diseases, which lack appropriate research and evaluation in these regions. As a result, the actual GBD of occupational ergonomic factor-induced LBP may be underestimated.

To address these challenges, we propose a tiered intervention approach beginning with short-term priorities including targeted ergonomic audits in low-SDI regions and integration of rapid assessment tools into primary healthcare systems, followed by medium-to-long term strategies such as implementing evidence-based telework regulations requiring employer-provided ergonomic subsidies and biannual evaluations, while establishing regional occupational health centers to distribute low-cost protective equipment and standardized training. Future research should focus on evaluating the long-term impact of changes in working patterns post-COVID-19 on spinal health. Furthermore, to develop SDI-specific risk prediction models that holistically assess combined occupational and domestic ergonomic exposures. These measures will help compensate for the limitations of the current GBD assessment and provide a foundation for formulating more inclusive occupational health policies.

## Conclusion

In summary, the global all-age YLDs of occupational ergonomic factor-induced LBP has demonstrated a steady increase, rising from approximately 10 million person-years in 1990 to 14.173 million person-years in 2021. Due to population growth and aging, YLDs are projected to increase to more than 15 million person-years in 2037 across all age groups. While the disease burden of occupational ergonomic factor-induced LBP shows a decreasing trend, the rate of decline remains minimal. Females, low SDI regions, and middle-aged and older adults (40–64 years) continue to be the main contributors to the global burden of occupational ergonomic factor-induced LBP. Occupationally induced LBP is a prominent public health problem that requires urgent attention because of the lack of global, comprehensive, effective and targeted prevention, intervention and treatment.

## Data Availability

The original contributions presented in the study are included in the article/Supplementary material, further inquiries can be directed to the corresponding authors.

## Footnotes

^1^ http://ghdx.healthdata.org/gbd-results-tool
